# Alpha-Secretase ADAM10 Regulation: Insights into Alzheimer’s Disease Treatment

**DOI:** 10.3390/ph11010012

**Published:** 2018-01-29

**Authors:** Rafaela Peron, Izabela Pereira Vatanabe, Patricia Regina Manzine, Antoni Camins, Márcia Regina Cominetti

**Affiliations:** 1 Department of Gerontology, Federal University of São Carlos, São Carlos 13565-905, Brazil; rafaelaperoncardoso@gmail.com (R.P.); izabelavatanabe1@gmail.com (I.P.V.); patricia_manzine@yahoo.com.br (P.R.M.); 2 Departament de Farmacologia, Toxicologia i Química Terapèutica, Facultat de Farmàcia i Ciències de l’Alimentació, Universitat de Barcelona, 08028 Barcelona, Spain; camins@ub.edu; 3 Biomedical Research Networking Centre in Neurodegenerative Diseases (CIBERNED), 28031 Madrid, Spain; 4 Institut de Neurociències, Universitat de Barcelona, 08035 Barcelona, Spain

**Keywords:** ADAM10, Alzheimer’s disease, regulation, treatment

## Abstract

ADAM (a disintegrin and metalloproteinase) is a family of widely expressed, transmembrane and secreted proteins of approximately 750 amino acids in length with functions in cell adhesion and proteolytic processing of the ectodomains of diverse cell-surface receptors and signaling molecules. ADAM10 is the main α-secretase that cleaves APP (amyloid precursor protein) in the non-amyloidogenic pathway inhibiting the formation of β-amyloid peptide, whose accumulation and aggregation leads to neuronal degeneration in Alzheimer’s disease (AD). ADAM10 is a membrane-anchored metalloprotease that sheds, besides APP, the ectodomain of a large variety of cell-surface proteins including cytokines, adhesion molecules and notch. APP cleavage by ADAM10 results in the production of an APP-derived fragment, sAPPα, which is neuroprotective. As increased ADAM10 activity protects the brain from β-amyloid deposition in AD, this strategy has been proved to be effective in treating neurodegenerative diseases, including AD. Here, we describe the physiological mechanisms regulating ADAM10 expression at different levels, aiming to propose strategies for AD treatment. We report in this review on the physiological regulation of ADAM10 at the transcriptional level, by epigenetic factors, miRNAs and/or translational and post-translational levels. In addition, we describe the conditions that can change ADAM10 expression in vitro and in vivo, and discuss how this knowledge may help in AD treatment. Regulation of ADAM10 is achieved by multiple mechanisms that include transcriptional, translational and post-translational strategies, which we will summarize in this review.

## 1. Introduction

ADAM (a disintegrin and metalloproteinase) is a family of transmembrane and secreted metalloproteinases comprising approximately 750 amino acids, with functions in cell adhesion and proteolytic processing of the ectodomains of diverse cell-surface receptors and signaling molecules [1]. APP (amyloid precursor protein) cleavage by ADAM10 results in the production of an APP-derived fragment, sAPPα, which is neuroprotective. Given that increased ADAM10 activity protects the brain from β-amyloid deposition, this strategy is viable in terms of treating neurodegenerative conditions, including Alzheimer’s disease (AD) [2]. We report in this review on the physiological regulation of ADAM10 at the transcriptional level, by epigenetic factors, miRNAs and/or translational and post-translational levels. In addition, we describe the conditions that can change ADAM10 expression in vitro and in vivo, and discuss how this knowledge may help in AD treatment.

It is worth mentioning that ADAM10 is ubiquitously expressed in mammalian cells and is involved in a series of other cleavages of cell-surface receptors and signaling molecules related to different normal and disease conditions. For this reason, any stimulation of this protein activity needs to be carefully investigated. Chronic or acute pharmacological stimulations of ADAM10 would engender many deleterious consequences, especially regarding its tumor-promoting activities [3].

## 2. Transcriptional Regulators

### 2.1. Retinoic Acid

**Figure 1:**
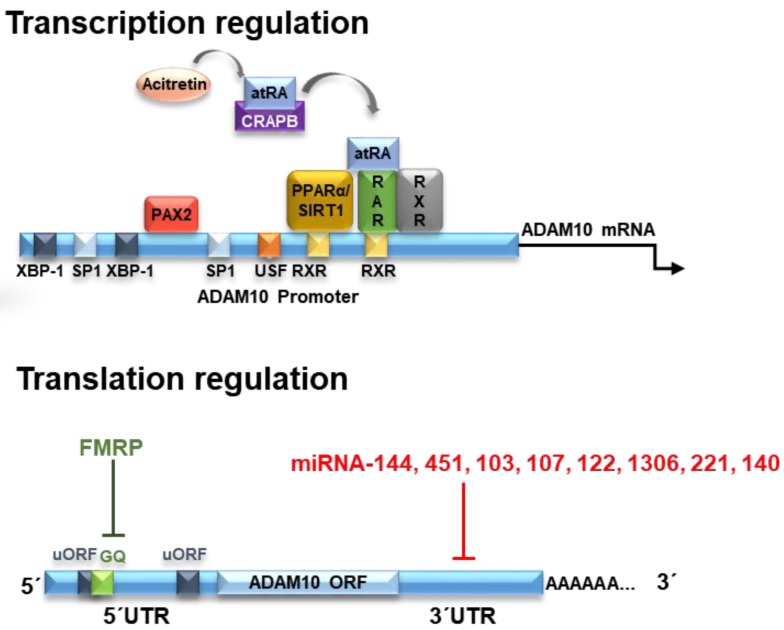
ADAM10 (a disintegrin and metalloproteinase 10) regulation at transcriptional and translational levels. Transcription of ADAM10 is regulated by various transcription factors. Its binding sites in the promoter region of ADAM10 are indicated by the colored squares. One of them is the RAR/RXR heteromer that can bind to the two RXR sites located in the ADAM10 promoter region. As a consequence of the binding of all-trans retinoic acid (atRA) in RAR, the RAR/RXR factor stimulates transcription of ADAM10. The acitretin drug, a derivative of retinoic acid, can remove atRA from retinoic acid-bound cellular protein (CRABP), leading to binding of atRA in RAR and stimulating the gene expression of ADAM10. The ADAM10 mRNA is formed by a GC-rich 5′UTR (untranslated region), the open coding structure (ORF) and the 3′UTR region. Two upstream open coding regions (uORF) are found in the 5′UTR region, but do not control the translation of ADAM10. On the other hand, a G-quadruplex (GQ) secondary structure inhibits translation of ADAM10 but may also be influenced by binding proteins such as FMRP. Likewise, different miRNAs inhibit the translation of ADAM10 by binding at different sites in the 3′UTR region. Extracted and modified from [8].

The human ADAM10 gene comprises 154 kb, it is composed of 16 exons and it is evolutionarily highly conserved (Figure 1). Nucleotides −508 to −300 were identified as the core promoter, and retinoic acid (RA) was identified as an inducer of human ADAM10 promoter activity [4]. RA is a metabolic product of vitamin A (retinol) which is not synthesized by animals, but obtained by diet [5]. Inside the cells, RA is metabolized to all-trans RA (atRA), however, it also occurs in various stereoisomeric forms including predominantly 13-*cis* RA and less-stable isomers such as 9-*cis* RA [6]. RA effects are mediated by its binding to nuclear retinoic acid receptors (RARs) and retinoid X receptors (RXRs) [7]. 

RA was demonstrated to transcriptionally upregulate ADAM10 mRNA levels, consequently stimulating the α-secretase process of APP and decreasing the amyloid-β formation. Nucleotides −508 to −300 bp are the core promoter on the ADAM10 gene [9] and two potential RA-responsive elements (RAREs) are located in the ADAM10 promoter region −302 and −203 bp upstream of the translation start site of the ADAM10 gene [9]. The binding of atRA or *cis*-RA to their respective cognate receptors RAR and RXR on the ADAM10 promoter then triggers the ADAM10 transcription [10]. Therefore, RA could be considered as having neuroprotective functions against AD.

Development of AD is accompanied by a large set of cellular and molecular events. Evidence shows that free-radical formation during oxidative stress is an early event in AD pathogenesis [11]. Vitamin A has been suggested to reduce the cellular oxidative stress and it is considered a potent antioxidant [12], and is proposed as a novel intervention for targeting AD’s early changes [13]. Thus, RA-induced stimulation of ADAM10 expression is likely to be physiologically relevant for further anti-AD therapy through the increase in APPα secretion and/or decrease of Aβ production in vitro [14,15], as well as in AD patients treated with acitretin, a vitamin A derivative [16]. Remarkably, to the best of our knowledge, the study of Endres and colleagues [16] was the only one reported in the literature demonstrating that a treatment with this synthetic vitamin A derivative is able to enhance non-amyloidogenic APP processing in human patients. In addition, it has been demonstrated that vitamin A deficiency leads to an increase in Aβ peptide levels in wild-type mice, and that the rescue of this deficiency increased non-amyloidogenic APP processing in combination with an increase of ADAM10 levels [17]. 

Another component of the RA pathway is the peroxisome proliferator-activated receptor (PPARα), as it interacts with RXR to form a heterodimeric structure that binds to RARs. PPARα is a transcription factor involved in fatty acid metabolism and it is constitutively expressed in the hippocampal neurons [18]. PPARα was demonstrated to activate ADAM10 transcription, reducing endogenous Aβ production by shifting APP processing toward the α-secretase pathway in vivo [19], showing that it is an important partner for retinoic acid to stimulate ADAM10 transcription [10].

Altogether, these data seem to point out that vitamin A supplementation would improve cognition and enhance AD behavioral and psychological symptoms. However, among the three clinical trials related to vitamin A supplementation in AD found in the clinical trial website, the only one reported in the literature demonstrated that participants only maintained, not improved, their baseline cognitive performance and behavioral and psychological symptoms of dementia over 12 months [20]. Whether other synthetic retinoid derivatives or therapeutic regimens will be able to increase cognitive performance in human patients remains to be seen.

### 2.2. Sirtuins

Sirtuins are a family of nicotinamide adenine dinucleotide (NAD+)-dependent deacetylases present in mammals. Specifically, SIRT1 has the ability to intervene and attenuate ageing-associated diseases, such as chronic inflammation, and metabolic, cardiovascular, neoplastic and neurodegenerative pathologies [21]. It was demonstrated that SIRT1 can act on ADAM10 transcription activation, increasing its expression and consequently reducing Aβ production [22] due to its ability to co-activate the retinoic acid receptor (RXR) leading to ADAM10 activation [23].

In transgenic animal models for AD, it has been shown that brain pathology and behavioral deficits have been minimized in animals expressing SIRT1, and exacerbated in brain-depleted SIRT1 animals. In addition, scientific evidence has shown that SIRT1 may also increase stress tolerance in AD brain neurons [24,25,26]. Various other studies are in accordance with these data, also reporting that SIRT1 induction by natural compounds such as resveratrol, or by metabolic conditioning linked to caloric restriction, have been shown to be important promising strategies to delay or even stop neurodegenerative processes [27,28,29].

### 2.3. XBP-1

The X-box binding protein (XBP)-1 positively regulates ADAM10 in neuronal cells [30]. More specifically, studies on AD transgenic animal models have shown that XBP-1 is linked to ADAM10 transcriptional regulation. XBP-1 is a transcriptional regulator activated by inositol-requiring enzyme 1-α (IRE1-α), an endoplasmic reticulum (ER)-stress sensor that specifically regulates the unfolded protein response (UPR). This transcription factor had a strong effect on the levels of ADAM10 and studies have shown that in AD patients, ADAM10 is reduced. Moreover, XBP-1 and its expression is dose-dependently regulated by XBP-1 and could be synergistically improved by insulin administration. Therefore, ADAM10 transcription is modulated by XBP-1 in neuronal cells by pharmacological stress induction of endoplasmic reticulum [31,32,33].

One of the XBP-1 transcription targets during ER stress is the protein reductase 1 (HRD1). Studies have demonstrated that HRD1 expression is decreased in AD brains. HRD1 is also colocalized and interacts with APP in brain neurons through proline-rich regions. Suppression of HRD1 expression induced APP accumulation, which in turn increased ER-stress-associated Aβ production. Furthermore, suppression of HRD1 expression inhibited APP aggresome formation, resulting in apoptosis of neuronal cells, a common event in AD [34]. Hence, in addition to its role in ADAM10 expression, through HRD1 activation and APP degradation, XBP-1 indirectly modulates Aβ production [35].

The ER homeostasis regulation is a fundamental characteristic in several pathological conditions. When UPR fails to decrease the ER stress imbalance, it induces cell death. This point is critical in neurodegenerative diseases as neuronal cell death is highly harmful [35]. In general, XBP-1 is a transcription factor that regulates a broad set of proteins involved in many functions linked or independent of ER stress and UPR, and therefore, can be seen as an important target for therapeutic strategies aiming to interfere with neurodegenerative pathologies such as AD [35].

### 2.4. Melatonin

Melatonin is a widely bodily distributed hormone, produced naturally endogenously, and responsible for controlling several physiological functions, including circadian rhythm regulation, clearance of free radicals and neuroprotection. Several observations have demonstrated the role of melatonin in the aging processes and AD progression. Firstly, melatonin has been demonstrated to clearly act as an agonist for ADAM10 transcription, acting directly on the promoter regions 1193 and 2304, and leading to ADAM10 increased expression [36,37]. Secondly, studies have shown that melatonin plasma levels strongly decrease with advancing age, and even lower levels are found in AD patients. Of particular note, melatonin loss in the cerebrospinal fluid (CSF) is parallel to AD neuropathological progression, and therefore melatonin levels are lower in patients with AD at the early stages [38,39,40,41,42]. Thirdly, melatonin has a great competence for metabolite elimination, which can be useful for Aβ elimination in AD [43,44,45]. Notably, in addition to its anti-amyloidogenic properties, melatonin has antioxidant roles since it inhibits Aβd aggregation, protects against Aβ-induced apoptosis and improves learning and memory deficits in transgenic mice models of AD [46,47,48].

Finally, it has been described that melatonin attenuates Bax, caspase-3 and par-4-dependent Aβ increase, and also reduces reactive oxygen species (ROS) accumulation [49,50]. Studies have shown that chronic administration of melatonin in AD animal models efficiently reduced Aβ brain accumulation [51,52,53]. Altogether, these studies demonstrate that melatonin activity promotes the inhibition of amyloid plaques and Aβ toxic species formation, inducing APP non-amyloidogenic processing by ADAM10 [36,37]. Previous studies report that this hormone is largely responsive to the ADAM10 promoter region. Among these promoters, Oct-1 (OC tamer-binding transcription factor-1), CREB (cAMP response element-binding protein) and HIF-1 (hypoxia-inducible factor-1) are important players [36]. Melatonin receptor stimulation on the plasma membrane can induce ERK phosphorylation through three distinct signaling pathways (Gq/PLC/PKC, Gi/PI3K/PDK1/PKC or Gs/cAMP/PKA), leading to transcription factor activation, including CREB, Oct-1 and HIF-1, and other ERK inducible factors that may also be involved. Interestingly, CREB, Oct-1 and HIF-1 are all under the positive control of ERK, thus they are also stimulated by melatonin and therefore induce ADAM10 transcription [36]. The same study also demonstrated that lower doses of melatonin could activate the HIF-1 and subsequently stimulate ADAM10 transcription [36]. 

On the other hand, a recent meta-analysis that evaluated seven studies (*n*  =  462) demonstrated that melatonin did not improve cognitive abilities of AD patients who received this hormone from 10 days to 24 weeks, showing only effects on prolonging total sleep time at night in these patients [54]. Furthermore, the chronic administration of melatonin in an AD mouse model efficiently reduced Aβ aggregates in the brain when started at early stages of the disease [51,52], but failed to exert positive effects when the treatment was initiated after Aβ deposition [53].

Overall, melatonin seems to be a neuroprotective agent and may represent a valuable therapeutic approach to prevent AD. However, it is clear that more studies involving melatonin supplementation in AD must be conducted in order to clarify its role in AD treatment and/or prevention.

### 2.5. SOX-2

SOX-2 (Y sex determination region (SRY)-box 2) is a regulatory component of the transcriptional nucleus of the network that maintains cell totipotency during the period of embryonic pre-implantation [55,56]. SOX-2 deficiency not only impairs neurogenesis, but also induces neuronal degeneration in mouse brains [56]. In addition, SOX-2 levels are strongly decreased in AD transgenic animal models, as well as in AD patients’ brains. Considering this, the idea that any decrease in SOX-2 levels could favor the AD pathology was strongly supported [57]. SOX-2, in addition to its well-established role in maintaining pluripotent cells, has been shown to participate in the homeostasis and regeneration of several adult tissues [58] and is expressed and functional in adult hippocampal neural stem cells [59].

Evidence of a role for SOX-2 in ADAM10 regulation is related to the fact that it induces both the catalytic activity of ADAM10 and its immunoreactivity through a mechanism of transcription stimulation [60]. It is noteworthy that ADAM10-dependent SOX-2 regulation is facilitated, as these two proteins colocalize in the subventricular-zone brain region of adult individuals [61].

SOX-2 also acts by increasing APP sequential and consecutive cleavages of γ- and β-secretases, triggering an overproduction of the intracellular domain of APP (AICD) and increasing the Aβ release, generating impaired neurogenesis, neurodegeneration and cerebral morphological failures [60]. As a result, the formation of plaques promotes phosphorylated Tau protein aggregation and accumulation, synaptic loss, neuroinflammation, neurodegeneration and neuronal death, followed by an onset of AD symptoms [8]. In addition, the reduction of SOX-2 acts on the decrease of ADAM10 transcription levels and thus its activation could probably be considered as a protective factor against AD [56,60,62,63,64]. Furthermore, SOX-2 interacts with a signaling glycoprotein (Wnt), which is possibly involved in the AD pathogenesis. The loss of Wnt signaling may lead to GSK3β activation, an enzyme involved in neuronal cell development, which in turn must interfere with Aβ deposition, catenin degradation, activation of apoptosis pathways and, finally, in the AD pathophysiology [65].

### 2.6. PAX2

Paired box gene 2 (PAX2) is a member of the PAX gene group. PAX2 is a transcription factor that regulates the expression of genes involved in cell proliferation and growth, apoptosis resistance and cell migration [66]. All PAX genes usually have a paired domain, which can bind to DNA in a specific way to function as transcription factors [67]. Studies have shown that PAX2 clearly acts as an agonist for the promotion of ADAM10 transcription. PAX2 acts directly on the promoter regions of 313 to 321, leading to increased ADAM10 transcription [68]. When normally expressed, PAX2 functions as a transcription factor and as an epigenetic regulator [67,69,70]. Using different cell systems, PAX2 was identified as an ADAM10 regulatory factor. In melanoma cells, chromatin immunoprecipitation and overexpression, as well as siRNA-mediated knockdown, have shown that PAX2 regulates ADAM10 expression [71]. PAX2 reduction via siRNA in A498 cells (renal carcinoma), EAhy (endothelial), T98G (glioblastoma) and SKOV3ip (ovarian carcinoma) revealed almost total ADAM10 protein loss [67]. Therefore, PAX2 appears to play an important role in ADAM10 expression, at least in cancer cells [72].

## 3. Translational Regulators

The human ADAM10 (mRNA) transcript is 4.4 kb in size. After being transcribed into mature mRNA, ADAM10 is subjected to several translational down- or upregulating effectors acting on ADAM10 protein expression. The analysis of ADAM10 mRNA shows evidence of a GC-rich segment located in the 5′UTR region formed by about 450 nucleotides. Interestingly, the presence of this region inhibits the translation of ADAM10, and its deletion causes a large increase in expression in human liver cells [73]. This effect of ADAM10 inhibition of translation by the 5′UTR region is due to the presence of a stable secondary structure of G-quadruplex (GQ) RNA comprising a 28 nucleotide-long G-rich sequence responsible for this suppression, reducing ADAM10 protein levels [73,74].

The RNA G-quadruplex prevents the formation or scanning of the pre-initiation complex, thereby blocking mRNA translation into proteins [10,75]. Derivatives of 1-methylquinolinium with incorporated aromatic groups can specifically bind to the G-quadruplex-forming sequence of ADAM10 mRNA. This binding prevents the formation of the inhibitory highly ordered RNA-G-quadruplex complexes and increases the translation of ADAM10, as occurs with the fragile X mental retardation protein (FMRP) [76,77] (Figure 1). The FMRP regulates neuronal RNA metabolism, and its absence or mutations leads to the fragile X syndrome (FXS) [77]. In human FXS fibroblasts, a dual dysregulation of APP and ADAM10 leads to the production of an excess of sAPPα leading to synaptic and behavioral deficits. Therefore, in FXS, the inhibition of ADAM10 activity reduces sAPPα levels, restoring translational control, synaptic morphology and behavioral plasticity. Thus, contrary to AD, the inhibition of ADAM10-mediated APP processing is crucial for healthy spine formation and function [77].

Mechanisms of translational regulation of ADAM10 levels include the action of microRNAs (miRNAs). miRNAs are molecules of approximately 21 noncoding nucleotides capable of regulating gene expression at the post-transcriptional level [78]. The miRNAs are processed from precursor molecules (pri-miRNAs), which are transcribed by RNA polymerase II from independent genes or represent introns in genes encoding proteins [79]. The miRNAs bind to the 3′UTR region of target mRNAs by base pairing, resulting in either mRNA degradation or translational inhibition (Figure 1).

More than 400 miRNA species have been identified in the human brain and it is estimated that this organ may contain more than 1000 miRNAs [80]. Evidence from research on AD suggests that alterations in the miRNA network may contribute to an increased risk of developing the disease [81,82]. It has been demonstrated that miR-144 and miR-122 can bind to ADAM10 mRNA and promote its regulation, as its superexpression caused a decrease in the levels of this protein [83].

Another miRNA possibly involved in the regulation of ADAM10 is miR-451, which together with miR-144 (miR144/451) may act to inhibit the expression of ADAM10 [84]. In partial agreement with these studies, we recently showed that miR-144, -374 and -221 are downregulated in the total blood of AD subjects and ADAM10 protein levels are significantly decreased upon transient overexpression of miR-221 in SH-SY5Y cells, but not altered after overexpression of miR-144-5p and miR-374, indicating the specificity of miR-221 in the regulation of ADAM10 levels [85].

A computational approach and experimental validation were used to suggest possible miRNAs that act in the regulation of ADAM10 expression. Three miRNAs (miR-103, miR-107 and miR-1306) were found to be AD related and have binding sites maintained for ADAM10 among species, with miR-103 and miR-107 showing significant overlap with the AlzGene database. In SH-SY5Y cells, these three miRNAs showed significant inhibitory activity on ADAM10 expression levels [86]. 

In a very recent study, miR-140-5p was elevated in AD postmortem brain hippocampus, and presents binding sites in both the ADAM10 and its transcription factor SOX-2 3′UTR, suggesting that miR-140-5p has a high regulatory control on ADAM10 and AD pathogenesis [87].

In short, it has been demonstrated that miRNAs 103, 107, 122, 144, 221, 451, 1306 and 140 regulate ADAM10 expression through 3′UTR interaction, suggesting the possibility to develop novel therapeutic strategies that may act on its expression and be useful in AD treatment.

## 4. Post-Translational Regulators

**Figure 2:**
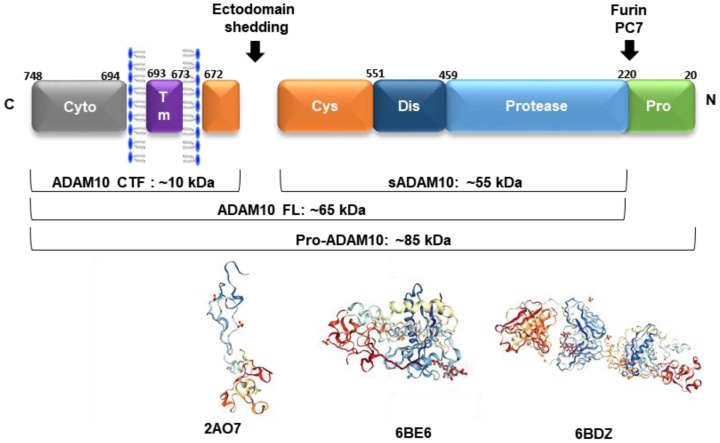
The ADAM10 multimodular structure (ADAM10 CTF: C-terminal fragment, sADAM10: soluble form, ADAM10 FL: full-length active form, pro-ADAM10: inactive protein with the presence of pro-domain). Below are represented the three-dimensional (3D) arrangements of the ADAM10 ectodomain (PDBs: 2AO7—disintegrin and cysteine-rich domains; 6BE6—extracellular domain and 6BDZ—extracellular domain bound by the 11G2 Fab), according to [89,90].

ADAM10 is a multimodular transmembrane protein ubiquitously expressed in mammalian cells, synthesized as an inactive 798 amino acid-long zymogen containing a C-terminal cytoplasmic (Cyto) domain, a transmembrane (TM) domain, a cysteine-rich domain (Cys, which can interact with cell-surface proteoglycans), a disintegrin domain (Dis, which binds to integrin cell adhesion molecules), a zinc-binding metalloprotease (Protease) domain, and a pro-domain (Pro, that is proteolytically removed by pro-protein convertases). Pro-ADAM10 has a molecular weight of ~85 kDa, and after pro-domain removal, the full-length (FL) ADAM10 has ~65 kDa. Ectodomain shedding leaves a ~10 kDa C-terminal fragment (CTF) membrane anchored, and releases a ~55 kDa soluble form (sADAM10) [88] (Figure 2).

The synthesis of proteinases as inactive zymogens is important to the cells because it allows them to spatially and temporally regulate proteolytic activities, thereby reducing the occurrence of premature enzymatic activities [91]. The 195 amino acid-long prodomain is important for folding and transport, and acts as a potent inhibitor of ADAM10 activity, maintaining the proteinase in a latent form via a cysteine switch mechanism. This mechanism is mediated by a highly conserved cysteine residue at position 173 in the prodomain, which interacts and neutralizes the zinc-coordinating HEXGHXXGXXHD catalytic core of the metalloprotease domain. In mammals, the enzymes responsible for many of these intracellular conversions are the proprotein convertases (PCs), which mediate the endoproteolytic processing of precursors, such as ADAM10. PC7 and furin are membrane-associated, calcium-dependent endoproteinases that proteolytically cleave several proproteins at the consensus sequence RX(K/R)R [91]. After the prodomain cleavage that occurs either in the trans-Golgi network or at the plasma membrane, ADAM10 becomes fully active and several mechanisms regulate its function from this point forward [10].

ADAM10 can also be subject to regulated intramembrane proteolysis. Other proteins from the ADAM family (ADAM9 and -15) act as proteases allowing the releasing of the ADAM10 ectodomain. On the other hand, γ-secretases can release the ADAM10 intracellular domain, enabling it to be translocated to the nucleus, which it is thought to be involved in gene regulation. Thus, ADAM10 performs a dual role in cells, as a metalloprotease when it is membrane-bound, and as a potential signaling protein once cleaved by ADAM9/15 and the γ-secretase [88]. This was reinforced by the study of Cissé and coworkers [92], who demonstrated that ADAM9 is unable to cleave a fluorimetric substrate of membrane-bound α-secretase activity in ADAM10-/-fibroblasts. However, the co-expression of ADAM9 and ADAM10 in ADAM10-deficient fibroblasts led to enhanced membrane-bound and released fluorimetric substrate-hydrolyzing activity when compared with that observed after ADAM10 cDNA transfection alone in ADAM10-/-cells.

In this review, we will focus on the components and pathways that activate ADAM10, rather than inhibitory mechanisms. These features enable ADAM10 post-translational regulation in several different forms, as described below.

### 4.1. Metallothioneins

ADAM10 prodomain has two PC recognition sequences (RKKR) at positions 210–213 [91] and 48–51 [93]. It was reported that metallothionein-3 (MT-3) can increase the expression of PC7 and furin, thereby playing a role in the generation of active ADAM10 [94]. Metallothioneins are low-molecular-weight, cysteine-rich proteins found in a wide range of species and involved in the regulation of transport, storage and transfer of zinc from various enzymes and transcription factors [95]. MT-3 is mainly expressed in the central nervous system and its levels are decreased in the brains of AD patients and animal models [96], despite the fact that this is not a consistent finding [97,98,99]. It seems that the protective role of MT-3 from AD pathology is related to the protection of neuronal cells against the toxic effects of Aβ peptide [100,101].

### 4.2. Cellular Trafficking Regulators: Tetraspanins, SAP97 and AP2

The regulation of transmembrane proteins by compartmentalization into membrane microdomains is well known. Tetraspanins (Tspans) function by interacting with specific ‘partner proteins’ regulating their intracellular trafficking and lateral mobility, and clustering at the cell surface. This network of interactions is referred to as tetraspanin web. Tspans were described as ADAM10 partners [102] controlling its intracellular trafficking and clustering at the plasma membrane [103,104], demonstrating that the interaction of this secretase with other proteins is a key mechanism responsible for its regulation [105]. In fact, ADAM10 is one of the most commonly identified tetraspanin-associated proteins in proteomic studies, and the majority of ADAM10 appears to be tetraspanin-associated [106].

The TspanC8 subgroup of tetraspanins consists of Tspans 5, 10, 14, 15, 17 and 33. TspanC8 was identified as a regulator of ADAM10 maturation and trafficking in multiple cell types and species. Specifically, TspanC8 regulates the ADAM10 exit from the ER and transport to the plasma membrane, and removes its inhibitory prodomain, promoting its maturation [107,108,109]. Different TspanC8s interact with distinct regions of the ADAM10 extracellular region, suggesting that this secretase can adopt different conformational complexes with different TspanC8s, and that different TspanC8s can promote or inhibit ADAM10 cleavage of distinct substrates. These findings led to the hypothesis that ADAM10 can no longer be regarded as one molecular scissor, but instead, exists as six different scissors with different substrate specificities, depending on which TspanC8 it is associated with [109]. The ability of TspanC8 to interact with ADAM10 may enable targeting of these molecules in order to modulate the ADAM10 functions in AD in a tissue- or substrate-restricted manner.

Synapse-associated protein 97 (SAP97) is a member of the membrane-associated guanylate kinase family of proteins that are primarily responsible for structural organization in glutamatergic synapses [110]. ADAM10 has a synaptic relationship with SAP97, and this interaction allows ADAM10 recruitment to the synaptic membrane regulating its activity. The homology domain SRC (SH3) of SAP97 binds to proline-rich Pro-Lys-Leu-Pro motif of the ADAM10 within the cytoplasmic tail, thereby leading the protease to the postsynaptic membrane, enhancing α-secretase cleavage [2,111].

The phosphorylation of SAP97 has important implications for ADAM10 activity. Activation of PKC positively modulates the ADAM10 association to SAP97 [112]. Interruptions disrupting the ADAM10/SAP97 complex in rodents led to a reduction of ADAM10 localized at postsynaptic sites and a change in APP metabolism [111]. SAP97 was described as a determinant in ADAM10 enzyme activity, and modifications of SAP97 in AD pathogenesis could lead to ADAM10 reduction in the postsynaptic membrane, as it was demonstrated that ADAM10/SAP97 interaction is reduced in the hippocampus of AD patients [113]. Saraceno et al. [112] observed a significant reduction in the SAP97 phosphorylation in AD patients, which may be responsible for the reported defects in the ADAM10 trafficking and synaptic activity described by Marcello et al. [113].

Endocytosis is also an important pathway for ADAM10 synaptic regulation. Endocytosis is mediated by the clathrin protein and clathrin-adapting protein (AP2), which interact with an atypical region of the intracellular domain of ADAM10. This association favors the internalization of these enzymes, and therefore, negatively influences their expression and activity as α-secretase [114]. Studies in patients’ brains show that the AP2–ADAM10 interaction is increased in AD subjects compared to cognitively healthy subjects [115].

### 4.3. Acetylcholinesterase Inhibitors (AChEIs)

The treatment of AD is basically made with acetylcholinesterase inhibitors (AChEIs) [116]. Donepezil (1-benzyl-4-[(5,6-dimethoxy-1-oxoindan-2-yl)methyl]piperidine hydrochloride) is a specific and potent AChEI that has been shown to shift APP metabolism towards the non-amyloidogenic pathway and also to promote ADAM10 trafficking to the plasma membrane, enhancing α-secretase activity in vitro [117]. Whether other AChEIs act on increasing ADAM10 activity remains to be investigated. On the other hand, a recent study reported that six months of AChEI treatment does not significantly increase ADAM10 levels, but reduces BACE1 levels in AD platelets [118]. However, according to the data we found in our group, the prolonged use of this medication seems to substantially increase ADAM10 protein expression in platelet lysates of AD subjects (unpublished results), as well as the treatment with serotoninergic antidepressants [119]. In agreement with our observations, other groups also reported on the beneficial effects of antidepressants either in animal models [120] or in AD patients [121].

### 4.4. Natural Products

Some natural compounds have shown to act on α-secretase activation. These include curcumin, a natural component extracted from the plant *Curcuma longa* that presents anti-inflammatory, antioxidant, and copper and iron chelation properties. This natural product, due to its size, can easily penetrate the blood–brain barrier (BBB), and was suggested as a promising therapy for AD [122]. Curcumin conjugated with isoleucine, phenylalanine or valine at both extremities—but not curcumin alone or its metabolite tetrahydro-curcumin—was able to enhance ADAM10 protein expression and sAPPα secretion in vitro [123]. The mechanisms underlying these effects are still unclear, however it seems that curcumin can activate the expression of SIRT1 [124], transcriptionally increasing ADAM10 expression [15].

Gingerol is a dietary compound found in several plants belonging to the Zingiberaceae family. [6]-Gingerol is the major phenolic constituent of ginger and was reported to have antitumor, antimutagenic, anti-apoptotic, antioxidant, anti-inflammatory, and cardio- and hepatoprotective effects [125]. In cultured PC12 cells, [6]-gingerol exhibited protective effects on Aβ1-42-induced apoptosis by reducing oxidative stress and inflammatory responses, suppressing the activation of GSK-3β and enhancing the activation of Akt, thereby exerting neuroprotective effects [126]. Moreover, [6]-gingerol was able to suppress Aβ25-35-induced intracellular ROS accumulation and restored Aβ25-35-depleted endogenous antioxidant glutathione levels. Furthermore, [6]-gingerol treatment was able to upregulate protein and mRNA of the antioxidant enzymes γ-glutamylcysteine ligase (GCL) and heme oxygenase-1 (HO-1) in SH-SY5Y cells. The expression of these enzymes seemed to be mediated by activation of NF-E2-related factor 2 (Nrf2), suggesting that this natural compound exhibits preventive and/or therapeutic potential for AD treatment [127].

Resveratrol (RSV) is a natural polyphenolic flavonoid, which can be found in grapes and red wine, and exerts neuroprotective and antioxidant properties [128]. RSV decreases total cholesterol concentration in hypercholesterolemic rats [129]. The direct and positive effect of RSV on AD pathology is related to the activation of nuclear retinoic acid receptors, which may activate ADAM10 gene transcription [9], as discussed earlier in this review. The treatment with RSV under experimental conditions in CHO (chinese hamster ovary) cells expressing human APP695 containing a Swedish mutation showed a significant increase in ADAM10 expression, especially its mature form, and may be the reason for the increase in the formation of the AβPP α-CTF fragment after RSV treatment [130].

Acetyl-l-carnitine (ALC) is a compound that helps to maintain mitochondrial bioenergetics and decreases the oxidative stress associated with aging [131]. ALC is present at high concentrations in the brain and contains portions of carnitine and acetyl, both with neurobiological properties. Carnitine is important in the β-oxidation of fatty acids and the acetyl portion can be used to maintain acetyl-CoA levels. Other reported neurobiological effects of ALC include brain energetic modulation and phospholipid metabolism, synaptic morphology and synaptic transmission of multiple neurotransmitters [132]. ALC is active in cholinergic neurons, where it is involved in the production of acetylcholine. ALC treatment has been shown to stimulate α-secretase activity and, consequently, to reduce the β-secretase-mediated pathway. It is known that pre-treatment of cortical neurons in culture with ALC significantly reduces Aβ-induced cytotoxicity, protein oxidation and lipid peroxidation in a concentration-dependent manner [131]. In the hippocampal neurons, treatment with ALC caused an increase in the level of ADAM10 in the post-synaptic compartment [133,134,135]. Another study showed that ALC can influence the non-amyloidogenic metabolism of APP, without affecting the total APP and ADAM10 levels. The data suggest that ALC did not alter the level of ADAM10 protein, but rather influenced the delivery of ADAM10 to the post-synaptic compartment, and consequently positively modulated its enzymatic activity towards APP in neuroblastoma cells [136].

### 4.5. Statins

Apolipoprotein E (ApoE) is a major cholesterol carrier that functions as a lipid transporter and helps injury repair in the brain. ApoE is a 34 kD glycoprotein that can be found in isoforms apoE2, apoE3 and apoE4 in humans, which are codified by the ε2, ε3 and ε4 alleles, respectively. Individuals carrying the ε4 allele are at increased risk of AD compared with those carrying the more common ε3 allele, whereas the ε2 allele decreases AD risk. ApoE lipoproteins bind to several cell-surface receptors to deliver lipids, and also to hydrophobic Aβ peptide, regulating Aβ aggregation and clearance in the brain, contributing to its metabolism [137]. Immunohistological evidence demonstrates that ApoE is codeposited in senile plaques in the brains of AD patients [138]. ApoE ε4 carriers have more abundant Aβ deposition in the form of senile plaques compared to non-carriers [139]. Recently it has been demonstrated that ADAM10 expression and activity are altered in AD, which can be influenced by the ApoE genotype [140]. ADAM10 levels are especially diminished in individuals with an APOE4 genotype, and its activity is reduced in the presence of APOE4 compared to the other APOE (APOE2 > APOE3 > APOE4) isoforms [140]. 

In addition to the presence of the APOE4 genotype, dysregulation of cholesterol metabolism in the brain has been associated with the AD pathogenesis [141,142], and high blood cholesterol concentrations were found in AD patients [143], together with an increase in the AD risk in later life [144]. Elevation of cholesterol decreases ADAM10 levels and is one of the factors that may increase the formation of insoluble Aβ42 [130]. On the other hand, cholesterol depletion below a critical concentration (about 60% of the initial amount) favors the increased enzymatic activity of ADAM10, along with increased membrane fluidity [145].

Corroborating these data, it has been described that statins—competitive inhibitors of 3-hydroxy-3-methylglutaryl (HMG)-CoA reductase, the enzyme that catalyzes the rate-limiting step in cholesterol biosynthesis [146]—may regulate the ADAM10 activity, due to the latter ability to lower cholesterol levels [147]. In-vitro cell-culture studies have demonstrated that cholesterol reduction by statins increased the formation of sAPPα, and reduced Aβ production [145,148]. In fact, treatment of human subjects for three months with lovastatin resulted in a decrease of Aβ peptides in serum [149], but clinical studies failed to confirm the effect of statins on Aβ levels in the brain [150]. Therefore, the positive influence of cholesterol modulation and/or statin administration on the ADAM10 activity in the central nervous system in vivo needs to be confirmed.

## 5. Conclusions

Increased interest in the ADAM10 function as an α-secretase acting on the non-amyloidogenic pathway of AD has recently been expressed. Taken together, the data from this review show that ADAM10 is controlled in a very complex manner at transcriptional, translational and post-translational levels. Whether interventions on ADAM10 regulation at different levels would provide better clinical outcomes for AD patients remains to be carefully investigated and tested, firstly in animal models and later, if promising, in clinical trials. This investigation, however, must consider the various other ADAM10 substrates, because although its activation would result in beneficial outputs for AD patients, it can result in dangerous triggers for other diseases, such as cancer. This is especially important, considering that ADAM10 is also associated with tumor progression, metastasis and inflammation by site-specific cleavage of several adhesion molecules and cytokines. Taking into account that all pharmacological treatments on AD have failed so far, it is timely and of utmost importance to identify new and specific pathways that can serve as the basis for novel therapies. Thus, the best knowledge on how ADAM10 is regulated will be useful to understand how to efficiently control its activity, both in physiological and pathological conditions. In summary, ADAM10 is clearly a promising therapeutic target for a wide range of diseases, but because of the positive and negative effects of ADAM10 in health and disease processes, substrate-specific ADAM10-targeting in AD may be necessary to avoid toxic side effects.
